# Study on Wastewater Demulsification Technology of Crude Oil in Xinjiang Oilfield

**DOI:** 10.3390/molecules28062873

**Published:** 2023-03-22

**Authors:** Jingui Ma, Liqiang Ma, Yongdi Gao, Yue Qin, Zhihao Jiao, Ruibo Guo, Junwei Hou

**Affiliations:** 1 State Key Laboratory of Heavy Oil Processing, School of Engineering, China University of Petroleum-Beijing at Karamay, Karamay 834000, China; 2 Shanghai EHS Value Environment Technology Co., Ltd., Shanghai 202150, China; 3 Zhoushan High Tech Industrial Zone Development and Construction (Group) Co., Ltd., Zhoushan 316000, China; 4 CNCP Auditing Service Center Company Limited, Beijing 100028, China; 5 PetroChina Kalamay Petrochemical Company, Karamay 834000, China

**Keywords:** water flooding, chemical flooding, fracturing backflow fluid, process optimization, demulsification

## Abstract

The Second Oil Production Plant of Xinjiang Oilfield produces a large amount of highly emulsified crude oil, which has a serious impact on the subsequent oil–water separation. At present, the concentration of demulsifier has increased to 2000 mg/L, but the demulsification effect is still poor. In this paper, the source and physical properties of highly emulsified crude oil are investigated firstly. The results show that highly emulsified crude oil is composed of three kinds of liquid: (1) conventional water flooding (WF); (2) chemical flooding (CF); (3) fracturing backflow fluid (FB). Among them, high zeta potential, low density difference, high viscosity, and small emulsion particles are responsible for the difficulty in the demulsification of the WF emulsion, while the high pH value is the reason why the CF emulsion is difficult to demulsify. Therefore, systematic experiments were implemented to investigate the optimal demulsification approach towards the three liquids above. As for the WF emulsion, it was necessary to raise the temperature to 70 °C and the concentration of the demulsifier to 200 mg/L. Moreover, it was only necessary to add 200 mg/L of demulsifier to break the CF emulsion after adjusting the pH value to 7, while no extra treatments were needed to break the FB emulsion. We hope this study can provide a new insight for the treatment of emulsions in the later stage of oilfield development.

## 1. Introduction

Highly emulsified crude oil is named for its strong emulsification, which is characterized by high water content, long standing time, and no demulsification, so it has a great impact on the subsequent treatment of crude oil. The thin-oil treatment station in the Second Oil Production Plant of Xinjiang Oilfield mostly produces highly emulsified crude oil with 66 mPa·s viscosity (30 °C) and 45% water content. The conventional demulsification process for this crude oil is raising the temperature to 40 °C and adding 2000 mg/L of demulsifier, which is complicated and overconsuming, so a cheap and simple demulsification and water separation method is urgently needed.

(1) $$v=\frac{2\;\left(\rho -\rho^{\prime }\right)gr^{2}}{9\eta }$$

According to the Einstein–Stokes Equation (1), there are many factors affecting demulsification [1,2,3,4,5,6,7,8,9,10,11,12], such as temperature, oil–water density difference, viscosity, suspended solids content, surfactant concentration, pH value, interfacial tension, and Zeta potential, etc. According to the thermochemical demulsification process, the molecular motion of the oil–water interface of the emulsion is strengthened with the increase in temperature, which is beneficial for the demulsification as a result. Cong et al. [13] selected two kinds of demulsifiers (SP169 and AR36) commonly used in an oilfield to implement thermochemical demulsification research. The experimental results showed that: (1) The optimal mass fraction of the water-soluble demulsifier SP169 is 150 μG·g^−1^ because the phase transition point of crude oil gradually moves forward with the addition of the water-soluble demulsifier and the increase in concentration, and the demulsification effect is first suppressed and then increased, which is also greatly affected by temperature. (2) The best mass fraction of the oil-soluble demulsifier AR36 is 100 μG·g^−1^. Due to the addition of the oil-soluble demulsifier, the phase transition point of crude oil gradually moves backward with the increase in concentration, and the demulsification effect increases first and then decreases, but the demulsification effect is less affected by temperature compared to the water-soluble demulsifier SP169.

As for the treatments of heavy oil, the high viscosity would cause difficulty in demulsifying, slow the molecular diffusion, and stabilize the boundary facial mask. Hou et al. [14] put forward a new approach for the thermochemical demulsification of high-viscosity heavy oil (HYW line, 120,000 mPa·S) after blending. Firstly, a kind of low-viscosity oil (SE line) with a viscosity of 640 mPa·S and water cut of 90% was selected as the blended oil. After investigating the viscosity of the SE line and HYW line at different temperatures after being fully blended, the results showed that the heavy oil blended model was in good fit with the Bingham model. When the temperature was 40 °C and the content of the SE line was 30%, the viscosity was less than 10,000 mPa·S, and the viscosity continued to decline with the increase in temperature. When the temperature exceeded 80 °C, the viscosity was less than 1000 mPa·S and the final design SE line content was 30%. The optimal demulsification parameters were optimized as 70 °C with a demulsifier concentration of 160 mg/L, which could demulsify the heavy oil by more than 90% in the practical experiments.

Suspended particles also have a great impact on the stability of the emulsion, which are closely arranged on the interface layer of the emulsion and form a “Pickering” emulsion, greatly enhancing the stability of the emulsion [15]. Thermochemical demulsification, electrochemical demulsification [16], and ultrasonic demulsification are mostly applied to treat this emulsion. According to the discharge and treatment requirements of a typical refinery, Zhang et al. studied the fluctuation of desalting wastewater quality [17].

According to the characteristics of desalination wastewater, its stability was studied, and the actual desalination wastewater was treated with tubular electrocoagulation technology for the first time. When the desalination device was backwashed, the oil content of the desalination effluent was higher than 400 mg/L, and the highest concentration was 1700 mg/L, which was obviously beyond the design value. The average value of COD was about 4484 mg/L, which is closely related to the properties of crude oil. The average dissolved COD was about 765 mg/L, mainly from salt-free feed water. Resin and asphaltene in backwashing wastewater accounted for 28% of the total oil content, which was higher than that of normal desalination wastewater. The spontaneous demulsification of desalination wastewater can occur, but it takes a long time. Aeration pretreatment for 30 min can reduce the COD of desalination effluent to 26.2% of the raw water, and electrocoagulation can further reduce the COD significantly. When the initial current was 1.0 A and the reaction time was 15 min, the average removal rates of COD and dissolved COD were 80% and 50%, respectively. The pseudo-first-order kinetic model is suitable for describing the removal of COD and dissolved COD in wastewater. Under this condition, the total operating cost is 0.92 CNY/m^3^. Yu et al. [18,19] studied the effects of ultrasonic intensity, action radius, action time, and temperature on the demulsification effect and compared it with the demulsification effect of field agents. Under the experimental conditions, the produced fluid of heavy oil was taken as the treatment object, the optimal ultrasonic amplitude was 40 μm, the action radius was 40 mm, and the action time was 80 s. This led to overemulsification with a larger ultrasonic amplitude and action radius, which is not conducive to demulsification. The dehydration rate decreased rapidly when the radius exceeded 40 mm. The dehydration effect increased with the increase in temperature. The optimal action time and action intensity should be selected according to oil properties. The crude oil before and after ultrasonic demulsification was characterized with a microscope. Compared with a single demulsifier, the ultrasonic–demulsifier-coupling-enhanced demulsification could increase the dehydration rate by about 15–20%, and the treatment effect of this technology is also competent for medium oil.

Surfactants and interfacial tension mainly affect the stability of a chemical flooding emulsion. Researchers have found that the surfactant concentration is high, the interfacial tension is low around the injection well, and the main emulsion is an oil-in-water emulsion [20,21,22]. With the continuous promotion of the oil displacement agent, the surfactant is diluted with formation water and adsorbed by the formation, and the content is greatly reduced. Moreover, the conversion from oil-in-water to water-in-oil occurs when the content of oil increases. Acid addition and a reverse demulsifier are generally applied for treating this type of emulsion. The pH has an impact on the existence of naphthenic acid, iron ion, and divalent cation in the solution, thus affecting the demulsification. Relevant research has shown that the water removal capacity increases with the increase in pH, and the water removal capacity reaches its peak when the pH is 7 (pure water) [5,6]. Obviously, the presence of hydrochloric acid increases the difficulty of the demulsification and dehydration of crude oil because the naphthenic acid in crude oil is activated, the number of emulsifiers is boomed, and the strength of the emulsification membrane is improved at low pH. In other words, the hydrogen ion concentration decreases with the increase in pH value and enhances the efficiency of crude oil demulsification as a result. The absolute value of zeta potential reflects the stability of the emulsion interface layer. The higher the absolute value, the stronger the stability. Relevant research on demulsification and dehydration processes coupled with ultrasonic and centrifugal technology has verified that the optimum deoiling rate is 92.46% when the centrifugal time is 20 min, the centrifugal speed is 4000 r·min^−1^, the ultrasonic irradiation time is 45 min, the ultrasonic settling time is 120 min, the ultrasonic power is 300 W, and the reaction temperature is 50 °C [23].

Different demulsification technologies and their characteristics are shown in Table 1. According to the requirements of the crude oil treatment station of Xinjiang Oilfield, the optimal scheme is screened: (1) the 2 h water separation rate in the laboratory reaches 90%; (2) according to the principle of “improving quality and efficiency” of Xinjiang Oilfield, the treatment temperature should not exceed 60 °C and the concentration of the demulsifier should be controlled within 200 mg/L. Therefore, we should find the best demulsification process according to the above conditions.

In this paper, we firstly analyzed the sources and the characteristics of each source of highly emulsified crude oil in the thin-oil treatment station of the Second Oil Production Plant. According to different characteristics, demulsification schemes were designed and tested. The results showed that highly emulsified crude oil comes from conventional water flooding, chemical flooding, and fracturing unconventional development, respectively. For a conventional water drive system, picking lotion formed by high suspended solids is the reason why demulsification is difficult to, and a demulsifier and temperature rise are required to act simultaneously to demulsify. For chemical flooding, the high pH value is the reason for its difficulty in demulsification, and only acid is needed for fracturing unconventional emulsification. Simple heating can break the emulsion.

## 2. Results

### 2.1. Basic Parameters

The basic physical properties of the WF, CF, and FB emulsions are shown in Table 2. It can be seen that the WF emulsion had the highest suspended solids content, up to 900 mg/L; CF had the highest pH, reaching up to 8.73. The chemical flooding used the ASP (alkali, surfactant, and polymer) ternary compound flooding formula. The injection concentration of sodium carbonate reached 12,000 mg/L, so the pH of the produced CF emulsion liquid was the highest. The produced water contained surfactants with a concentration of 304 mg/L. The mineralization degree of the FB emulsion was the highest, more than 21,000 mg/L, but the suspended solids and pH were relatively low.

### 2.2. Viscosity-Temperature Curve

**Figure 1:**
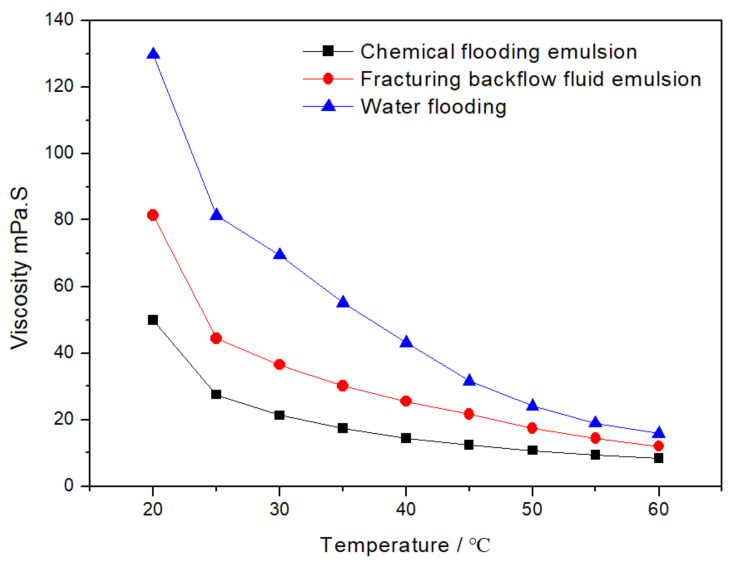
Viscosity–temperature curve of WF, CF, and FB emulsion.

Figure 1 shows the viscosity-temperature curve of WF, CF, and FB emulsion. It can be seen that the viscosity of the above three liquids is an inverse correlation to the temperature. The viscosity of WF, CF, and FB dropped to 20 mPa·s, 14 mPa·s, and 10 mPa·s, respectively, when the temperature increased to 60 °C. Furthermore, the viscosity of water flooding is the highest, reaching 130 mPa·s, while the initial viscosity of CF emulsion is only 51 mPa·s.

### 2.3. Interfacial Tension

**Figure 2:**
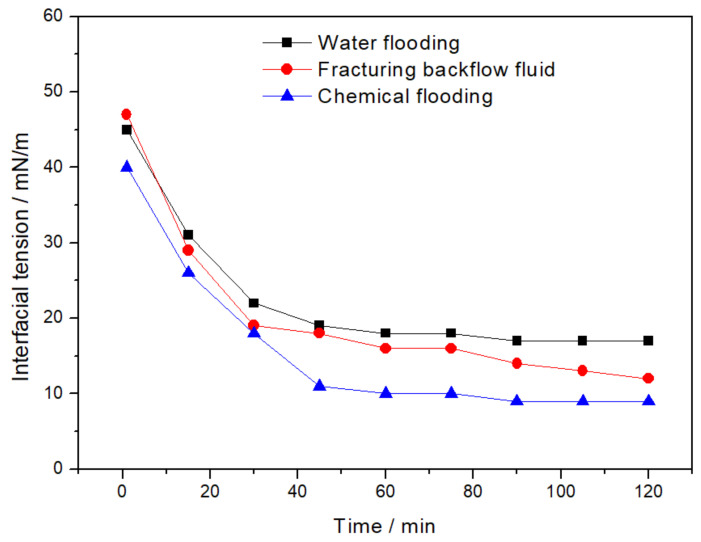
Interfacial tension of WF, CF, and FB emulsion.

Figure 2 shows the interfacial tension of the three kinds of fluids. It can be seen that the interfacial tension of the three fluids decreased rapidly with the extension of time and then became stable. In detail, the interfacial tension of WF emulsion was 45 mN/m at first, and it decreased rapidly and reached 18 mN/m after 120 min; the initial interfacial tension of the CF emulsion was the lowest, which was 40 mN/m, and decreased to 10 mN/m after 120 min; and the initial interfacial tension of the FB emulsion was 47 mN/m and decreased to 13 mN/m after 120 min.

### 2.4. Size and Type of Emulsion

**Figure 3:**
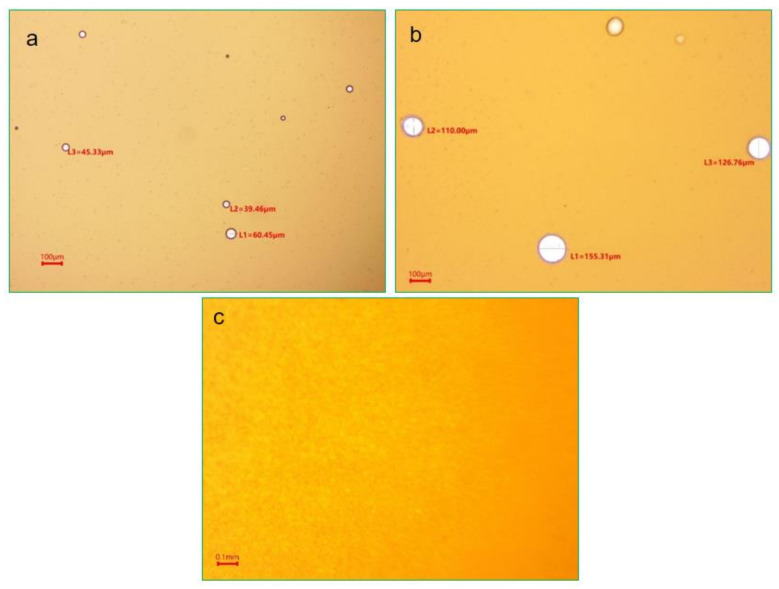
(**a**). The optical photos of WF emulsion; (**b**). CF emulsion; (**c**). FB emulsion.

Figure 3 shows the optical microscope photos of the WF, CF, and FB emulsions. It can be seen that the WF emulsion was mainly a water-in-oil emulsion with a diameter of 10–15 μm, while the CF emulsion was mainly a water-in-oil emulsion with a diameter of 30–50 μm (average diameter 40 μm). There was no emulsion in the FB, which may be because the demulsification was too fast and the crude oil and water were separated.

### 2.5. Oil–Water Density Difference

**Figure 4:**
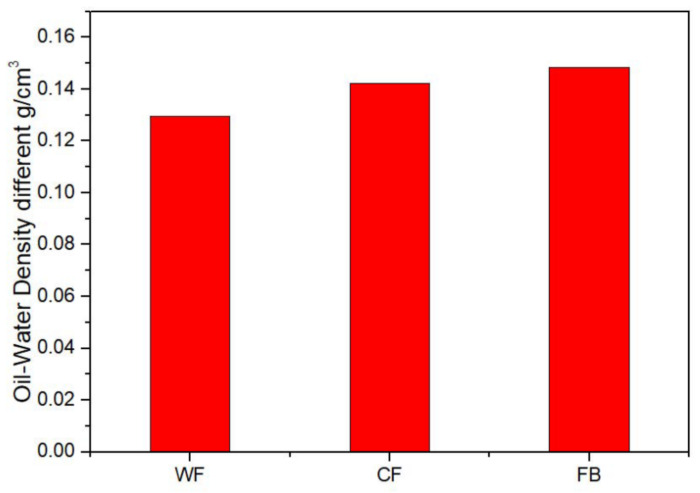
Oil–water density difference in WF emulsion, CF emulsion, and FB emulsion.

Figure 4 shows the oil–water density difference in the WF, CF, and FB emulsions. It can be seen that the oil–water density difference in the WF emulsion was the lowest, reaching 0.1296 g/cm^3^, while the oil–water density difference in the CF emulsion took second place, reaching 0.1423 g/cm^3^. The absolute value of zeta potential of the FB emulsion was the largest, reaching 0.1485 g/cm^3^.

### 2.6. Zeta Potential

**Figure 5:**
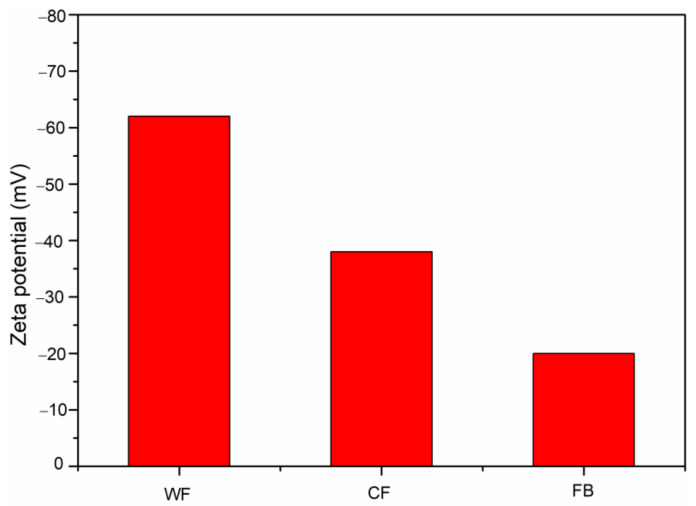
Zeta potential of WF emulsion, CF emulsion, and FB emulsion.

Figure 5 shows the zeta potential curve of the WF, CF and FB emulsions. It can be seen that the absolute value of zeta potential of the WF emulsion was the highest, reaching 62 mV. The absolute value of zeta potential of the CF emulsion took second place, reaching 38 mV. The absolute value of zeta potential of the FB emulsion was the smallest, only 20 mV.

### 2.7. Water Diversion Rate

**Figure 6:**
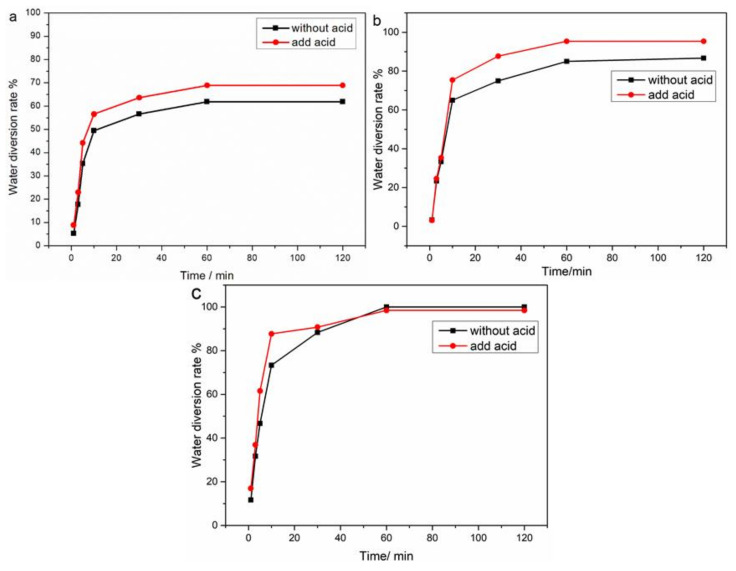
(**a**). The water diversion rate of WF emulsion; (**b**) CF emulsion; (**c**) FB emulsion.

Figure 6a shows the water diversion rate of the WF emulsion at different times when the concentration of the demulsifier was 200 mg/L, the temperature was 40 °C, no acid was added, and the concentration of hydrochloric acid was 0.1 M. It can be seen that the water separation rate gradually increased with the extension of time, and the final tax separation rate was only 61%. The effect of adding or not adding acid on the tax rate was very small. Figure 6b shows the water separation rate of the CF emulsion at different times when the concentration of the demulsifier was 200 mg/L, the temperature was 40 °C, there was no acid added, and the concentration of hydrochloric acid was 0.1 M. It can be seen that with the increase in time, the water separation rate gradually increased, the final water separation rate reached 81%, and the water separation rate after adding acid reached 92%, which proves that acid has a great impact on the stability of chemical flooding emulsion. Figure 6c shows the water separation rate of the FB emulsion at different times when the concentration of the demulsifier was 200 mg/L, the temperature was 40 °C, no acid was added, and the concentration of hydrochloric acid was 0.1 M. It can be seen that the FB demulsification was very fast, the demulsification rate reached 98% in 60 min, and the acid had no effect on the demulsification rate.

## 3. Discussion

### 3.1. Reasons for Emulsification

The WF emulsion had the highest suspended solids content, the highest absolute value of zeta potential, the maximum viscosity at 40 °C, the minimum emulsion size, the minimum density difference, the maximum interfacial tension, and the most difficult demulsification test. It is inferred that the WF emulsion is a “Pickering”-type emulsion formed by nanosolid particles surrounding the emulsion, which requires the combined action of the demulsifier and temperature rise before demulsification. The CF emulsion had the second-highest suspended solids content, the highest pH and surfactant concentration, the second-highest absolute value of zeta potential, the lowest interfacial tension, and the second-highest particle size in the emulsion. The demulsification test showed that the demulsification rate increased to 90% after adding acid. It is concluded that the emulsion is a common water-in-oil emulsion formed by a surfactant and alkali. The FB emulsion had the lowest suspended solids content, the lowest absolute value of zeta potential, and the highest interfacial tension. [24,25] The emulsion could be observed under the microscope. The test showed that it was very easy to demulsify, as can be seen in Table 3.

### 3.2. Comprehensive Demulsification Experiment of WF Emulsion

For emulsified oil with a small density difference, two methods can be selected. The first is to change the dehydration method, such as evaporation dehydration, centrifugal dehydration, and other dehydration methods that do not require the difference in oil and water density. The second is to select water-soluble demulsifying acids that can increase the water density and realize demulsification by increasing the density difference between oil and water. The crude oil density in the Wangji heavy oilfield is high. The dehydration problem is solved by adding water-soluble additives to increase the density difference between oil and water [26].

For emulsified oil with a high content of resin and asphaltene, a polyethylene polyamine polyoxyethylene polyoxypropylene ether double-block polyether demulsifier can be selected, which can absorb on the polar interface membrane, penetrate into the membrane, destroy the interface facial mask, and destroy the structure of resin asphaltene.

For emulsified oil with a high suspended solids content, FeS and naphthenate in suspended solids have a great impact on the stability of the oil–water interface facial mask. FeS is lipophilic and will accumulate on the oil–water interface during sedimentation, forming a rigid interface facial mask. Generally, an organic acid is selected to neutralize the charge. Sodium naphthenate is a typical emulsifier. Generally, the positive-ion treatment agent can neutralize the charge on the interface film and destroy the stability of the interface film. Among them, the effect of H^+^ is the best among the positive ions [27].

Finally, polyoxyethylene polyoxypropylene polyether with multiethylene polyamine as the initiator can be selected as the main agent, which is mixed with acetic acid in the ratio of 4:1 as the final demulsifier (A7).

**Figure 7:**
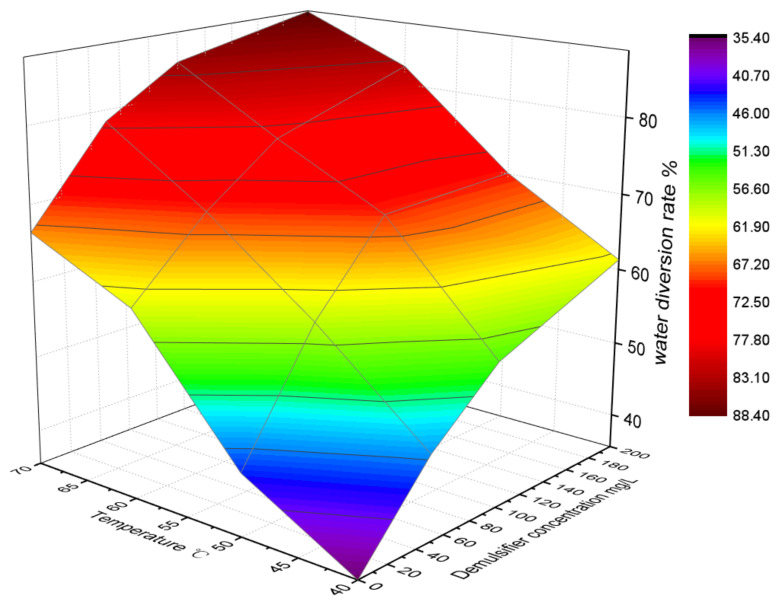
Effect of temperature and demulsifier concentration on WF demulsification.

Figure 7 shows the demulsification results of WF under the combined action of temperature and the demulsifier (A7). It can be seen that when the temperature was lower than 40 °C and the concentration of demulsifier was 0 mg/L, the demulsification rate in 120 min was only 34%. With the increase in temperature and concentration of the demulsifier, the demulsification rate gradually increased. When the temperature reached 70 °C and the concentration of the demulsifier reached 200 mg/L, the demulsification rate reached 90%.

## 4. Materials and Methods

### 4.1. Material and Reagent

We obtained the following materials: WF emulsion (No. 2 Oil Production Plant of Xinjiang Oilfield); CF emulsion (No. 2 Oil Production Plant of Xinjiang Oilfield); fracturing backflow fluid emulsion (No. 2 Oil Production Plant of Xinjiang Oilfield); A6 type cationic demulsifier for the oil treatment station (industrial product, Xinjiang Keli Company, Karamay, China); hydrochloric acid (Chemical pure, National Pharmaceutical Group); and A6 type cationic demulsifier. Chemical flooding contains two kinds of surfactant for oil displacement. One is Karamay petroleum sulfonate (KPS) anionic surfactant (industrial product, Xinjiang Jinta Company, Karamay, China). KPS is a petroleum sulfonate obtained from the sulfonation and alkali neutralization of aromatic distillate oil. Its composition and structure are complex, its molecular weight distribution range is from 300 to 550, and its main components are alkylidene, phenylhydrazine dicyclohexane, and alkylnaphthalene sulfonates. The other surfactant is alkanolamide nonionic surfactant (LPS, industrial product, Xinjiang Jinta Company, Karamay, China). The main component of the surfactant is N, N-dihydroxyethyl dodecyl amide.

The viscosity was tested with a rheometer (Antonpa MCR301, Anton Paar, Graz, Austria). The interface tension was tested with an interface tensiometer (TX-500C, Kono Industries Co., Ltd., Action, TX, USA). The zeta potential was tested by with zeta potentiometer (Malven Nano, Malvern Panalytical, Malvern, UK). The optical microscope photographs were tested with a microscope (AXIOSKOP 40, Carl Zeiss Optics, Oberkochen, Germany). The pH was tested with a pH meter (PHSJ-4F, Shanghai Yidian Scientific Instruments Co., Ltd., Shanghai, China). The demulsification experiment was conducted with a constant-temperature water bath system (HH-601A, Kanglu Scientific Instruments Co., Ltd., Beijing, China).

### 4.2. Experimental Method

**Figure 8:**
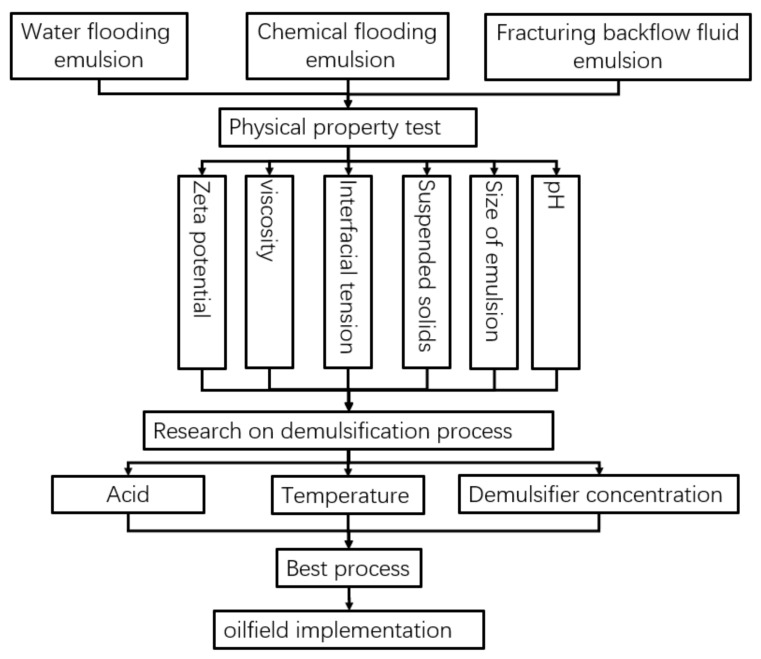
Experimental process of this study.

Firstly, the viscosity temperature curves at different blended ratios were measured using a Physica MCR301 rheometer. The temperature was 30 °C, 40 °C, 50 °C, and 60 °C, and the rotational speed was 100 s^−1^. Secondly, the size and type of emulsion were measured using a Zeiss AXIOSKOP 40 microscope. The emulsion interfacial tension was measured with a TX500C interfacial tensiometer with a rotating speed of 6000 rpm and temperature of 30 °C. The zeta potential of the emulsion was measured with a Malvern zetasizer nano at 30 °C. The test process is shown in Figure 8.

The water separation rate of the emulsion was measured using a 100 mL conical test tube and a constant-temperature water bath. The concentration of the added hydrochloric acid was 0.1 mol/L to adjust the pH of the emulsion to 7. The concentration of the demulsifier was 50 mg/L, 100 mg/L, and 200 mg/L, respectively. The volume *V*_1_ of the separated water was measured after 2 h, and the water content *V*_2_ of the mixed emulsion was measured with a water content meter; then, the water content met the following Equation (2).

(2) $$c=\frac{V_{1}}{V_{2}}\times 100\%$$

## 5. Conclusions

In conclusion, we firstly analyzed the properties of the highly emulsified crude oil in the Second Oil Production Plant and found that the highly emulsified crude oil is formed by the combination of water flooding, chemical flooding, and fracturing backflow. High zeta potential, low density difference, high viscosity, and small emulsion particles are the reasons for the difficulty in the demulsification of the WF emulsion. The scheme of a single line and single treatment is proposed. The high pH value is the reason why the CF emulsion is difficult to demulsify. The best demulsification process was studied, and the results showed that for WF emulsion, it is necessary to raise the temperature to 70 °C and the concentration of the demulsifier to 200 mg/L. For CF emulsion, it has the lowest interfacial tension and the highest pH value, so it is only necessary to add acid to adjust the pH value to 7 and add 200 mg/L of demulsifier to break the emulsion. For FB emulsion, it can be demulsified at 40 °C. We hope this study can provide a new insight for the treatment of emulsion in the later stage of oilfield development.

## Figures and Tables

**Table 1:** Different demulsification technologies and their characteristics.

Demulsification Technology	Region of Use	Characteristic
Blended oil	Xinjiang Oilfield, Shengli Field, and Liaohe Oilfield	Narrow application range and high control difficulty
Thermochemistry	Most oilfields	Excessive energy consumption
Ultrasonic	Saudi Aramco Oilfield and Bohai Oilfield	Narrow application range and high energy consumption
Evaporation technology	Liaohe Oilfield	Low energy consumption but high time cost
Biotechnology	Daqing Oilfield and Huabei Oilfield	Narrow application range and biological resistance
Microwave	Laboratory stage	Narrow application range and high energy consumption
Centrifugal technology	Ansai Oilfield, Caofeidian Oilfield, and Dagang Oilfield	Narrow application range and high energy consumption
Electrochemistry	Daqing Oilfield, Xinjiang Oilfield, and Nanhai Oilfield	Narrow application range and high energy consumption

**Table 2:** Analysis of water contents in WF, CF, and FB emulsion.

Name	Cl^−^ mg/L	KPS/LPS mg/L	Ca^2+^ mg/L	pH	HCO_3_^−^ mg/L	Mineralization Degree mg/L	Suspended Solids Content mg/L
WF	9827.42	0	146.6	7.45	983	17,637.2	900
CF	8062.4	304	678.16	8.78	4303.44	19,209.63	544
FB	12,529.42	0	1128	7.33	1098.67	21,911	276

**Table 3:** WF, CF, and FB emulsion performance comparison.

	WF Emulsion	CF Emulsion	FB Emulsion
Mineralization degree, mg/L	17,637.2	19,209.63	21,911
Viscosity at 40 °C, mPa·s	45	16	22
Interfacial tension, mN/m	18	10	13
Size and type of emulsion, μm	40	120	∞
pH	8.21	8.73	7.33
Zeta, mV	−62	−38	−20
Suspended solids content, mg/L	900	544	276
Density difference, g/cm^3^	0.1296	0.1423	0.1485
Water diversion rate, %	61%	86%	100%

## Data Availability

Data is contained within the article.

## Sample Availability

Samples of the compounds are available from the authors.
